# Supplementary material for: Central and peripheral nervous system complications of COVID-19: a prospective tertiary center cohort with 3-month follow-up
Source: J Neurol. 2021 Jan 13;268(9):3086–104. doi: 10.1007/s00415-020-10380-x (PMC7803470; doi:10.1007/s00415-020-10380-x)

**Fig. S1 – Screening tool used at bedside**

| **Social security number:___________________ Date:_____________**  **Form A: to be registered every day** **Day X__________**  *(If day 1 of admission, please fill out* ***form B****, if last day of admission please fill out* ***form C****)* | | | |
| --- | --- | --- | --- |
| **Consciousness levels** | | **Neurological symptoms** | |
| Coma due to sedation | Yes / No | Headache | Yes / No / Unknown |
| FOUR score (E:0-4,M:0-4,B:0-4,R:0-4) |  | Anosmia / Ageusia | Yes / No / Unknown |
| Pupils: Size & Light reflex (R/L) |  | Encephalopathy | Yes / No / Unknown |
| Cornea reflex | Yes, bilat. / Yes, unilat. / No | Seizures | Epileptic / non-epileptic / No |
| Oculocephalic reflex | Yes, bilat. / Yes, unilat. / No | Motor paresis | Hemi / Para / Tetra / 1 extremity / No |
| Grimace to pain | Yes / No | Abnormal movements | Hyperkinesia / Hypokinesa/ Ataxia / Terminal tremor / No |
| Cough reflex | Yes / No | Paresthesia | Localized / Universal / No / Unknown |
| **Respiration** |  | Suspicion of stroke | Yes / No |
| Intubation | Yes / No | Suspicion of medullary affection | Yes / No |
| Mechanic ventilation | Yes / No | Nystagmus | Yes / No |
| **Organ injury** | | **Psychiatric symptoms** | |
| Nephropathy | Yes, dialysis / Yes, no dialysis / No | Hallucinations | Yes / No / Unknown |
| Liver affection | Yes, clinical signs / Yes, biochemical signs / No | Paranoid | Yes / No / Unknown |
| Circulatory (MAP) | < 60 / 60-90 / >90 | Affect lability | Yes / No / Unknown |
| Circulatory (need for vasopressor agents) | Yes / No | Suspicion of delirium | Yes / No |
| **Level of treatment** | Full / Limited / Palliative care | **Other, describe** |  |
|  | | | |
| **Form B: Fill out if day 1 at the ward Day 1, date: ____________** | | | |
|  |  | **Previous medical history** | |
| Gender | Female / Male / Other | Somatic | HT / DM / Ischemic heart disease / Pulmonary / Nephropathy / Cancer |
| Ethnicity | Caucasian / Asian / Middle eastern / African / Greenland | Neurological | Stroke / Epilepsy / MS / Migraine / Dementia / Parkinson / other |
| Date of COVID symptom debut |  | Psychiatric | Depression / Bipolar / Schizophrenia / Anxiety / personality disorder |
| Date of COVID positive test |  | Immuno-suppression | Yes, leucopenia / Yes, chemo / Yes, other / No |
| Date of (ICU) admission |  | Baseline laboratory (WBC, CRP, krea) |  |
|  | | | |
| **Form C: Fill out at discharge from ward Date: ____________** | | | |
| **Clinical state** | Awake and oriented / Awake and disoriented / Unconscious | | |
| **Modified Rankin Scale** | 0 – Normal  1 – No significant disability. Able to carry out all usual activities, despite some symptoms.  2 – Slight disability. Able to look after own affairs without assistance, but unable to carry out all previous activities.  3 – Moderate disability. Requires some help, but able to walk unassisted. 4 – Moderately severe disability. Unable to attend to own bodily needs without assistance, and unable to walk unassisted. 5 – Severe disability. Requires constant nursing care and attention, bedridden, incontinent.  6 – Dead | | |

**Fig. S2**

A 77-year-old woman was admitted with 5 days of fever, malaise and progressive respiratory failure, with a positive SARS-CoV-2 PCR test on pharyngeal swab. She had a medical history of hypertension, hypothyroidism, cerebellar ischemic stroke and recurrent falls and had been investigated a year prior to admission as an outpatient with MRI of the brain (A1-A4). Coronal FLAIR (A1) showed severe leukaraiosis (Fazekas grade 3) and anterior temporal lobe hyperintensities (arrow), but no hyperintense lesions on diffusion-weighted imaging (DWI) (A2). T2*-weighted imaging showed few microbleeds in the basal ganglia (A3) and centrum semiovale (A4). She had mild cognitive decline, but was independent (modified Rankin Scale, mRS = 1). The patient stayed in the ICU for 40 days owing to COVID-19 induced respiratory failure. After withdrawal of sedation she had prolonged awakening and no verbal output or spontaneous movements, which lasted several days; however, during this time the patient could follow the examiner with her eyes. There were no focal deficits on examination, but cognitive deficits, which was interpreted as delirium, and general ICU-acquired weakness. MRI scan of the brain (B1) showed severe leukaraiosis (Fazekas grade 3) and cortical atrophy which was more severe than on the MRI a year earlier (A1). DWI showed hyperintense signal in the left corona radiata (B2), susceptibility-weighted imaging (SWI) revealed microbleeds in the basal ganglia (B3), and axial FLAIR showed anterior temporal pole hyperintensity (B4, arrow). The patient was transferred to the neurological department for rehabilitation. The family history was notable for several family members having cognitive decline at an early age, indicating cerebral autosomal dominant arteriopathy with subcortical infarcts and leukoencephalopathy (CADASIL). Genetic testing indeed revealed a known pathogenic heterozygote mutation in exon 1-25 of the NOTCH3 gene. The patient had initially been diagnosed with COVID-19 associated multi-infarction syndrome, a diagnosis of CADASIL was made following genetic confirmation of a relevant mutation. This case shows the diagnostic pitfalls of COVID-19 associated neurological complications and highlights the need clinical acumen and thorough work-up before neurological manifestations are attributed to COVID-19.

**Fig. S2**


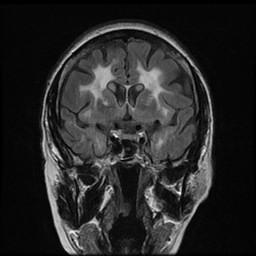


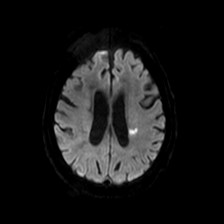


**B2**


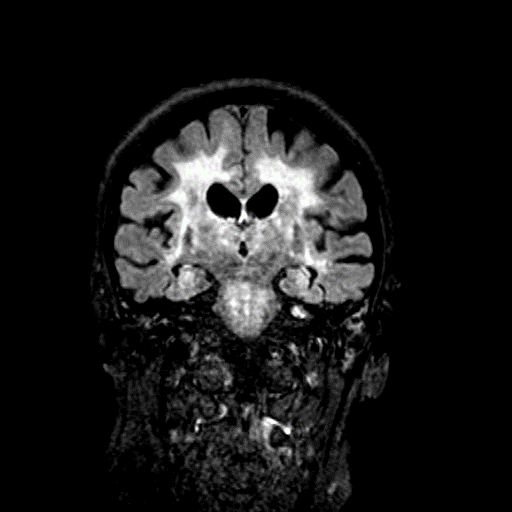


**B1**


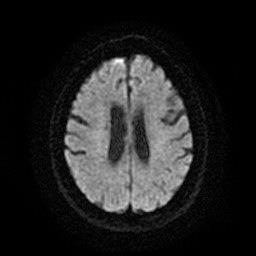


**A2**


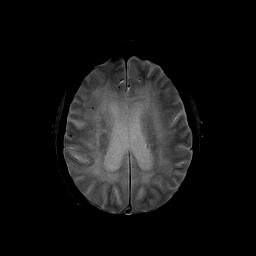

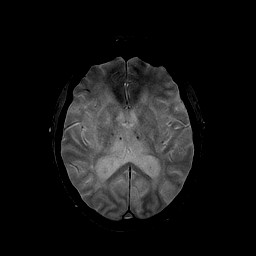

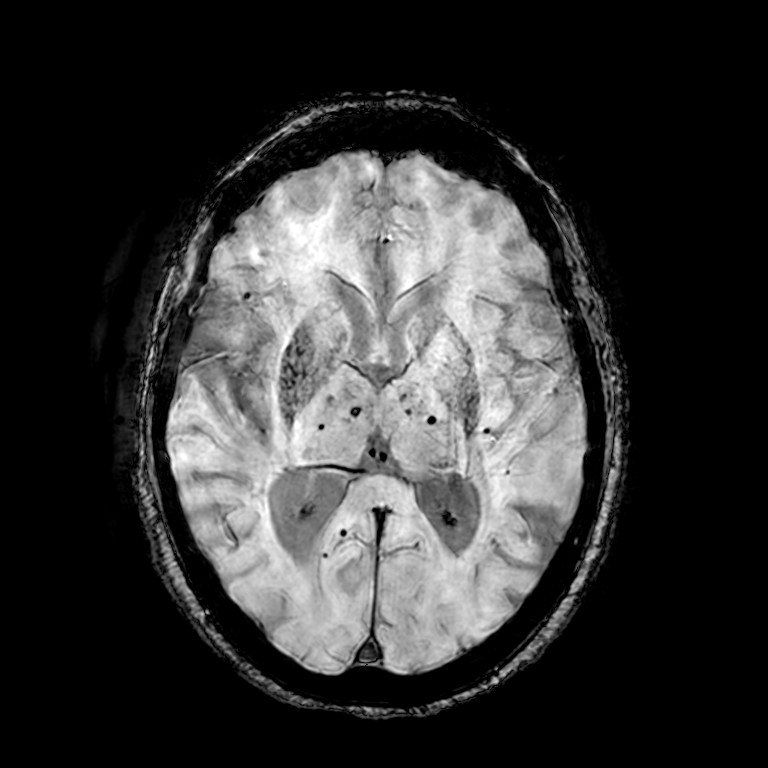


**B3**

**A3**

**A4**

**A1**



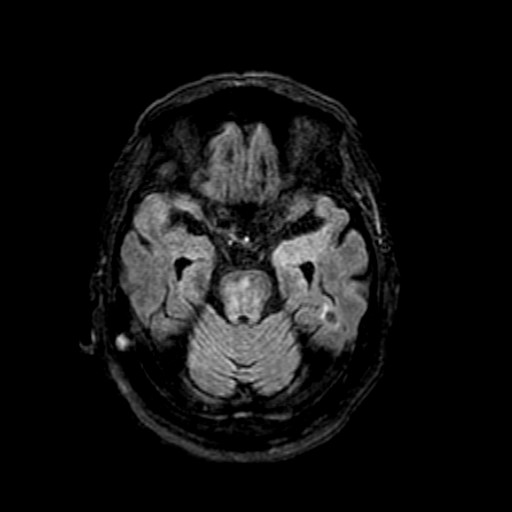


**B4**



**Fig. S3**

Figure legend: Detection of pre-specified neurological complications observed in 61 prospectively enrolled COVID-19 patients, hospitalized in a tertiary center from 1st of April to 25th of September. Day 0 represents onset of respiratory or systemic COVID-19 symptoms and marked symbol for each complication represents median of days to detection of specific complication when screened during the study period. Bars represent minimum and maximum range. True onset of specific complication may have been before detection by clinical screening. One patient may have several complications. CNS; Central nervous system. PNS; Peripheral nervous system. CIPM; Critical illness polyneuropathy or myopathy.

**Fig. S3**
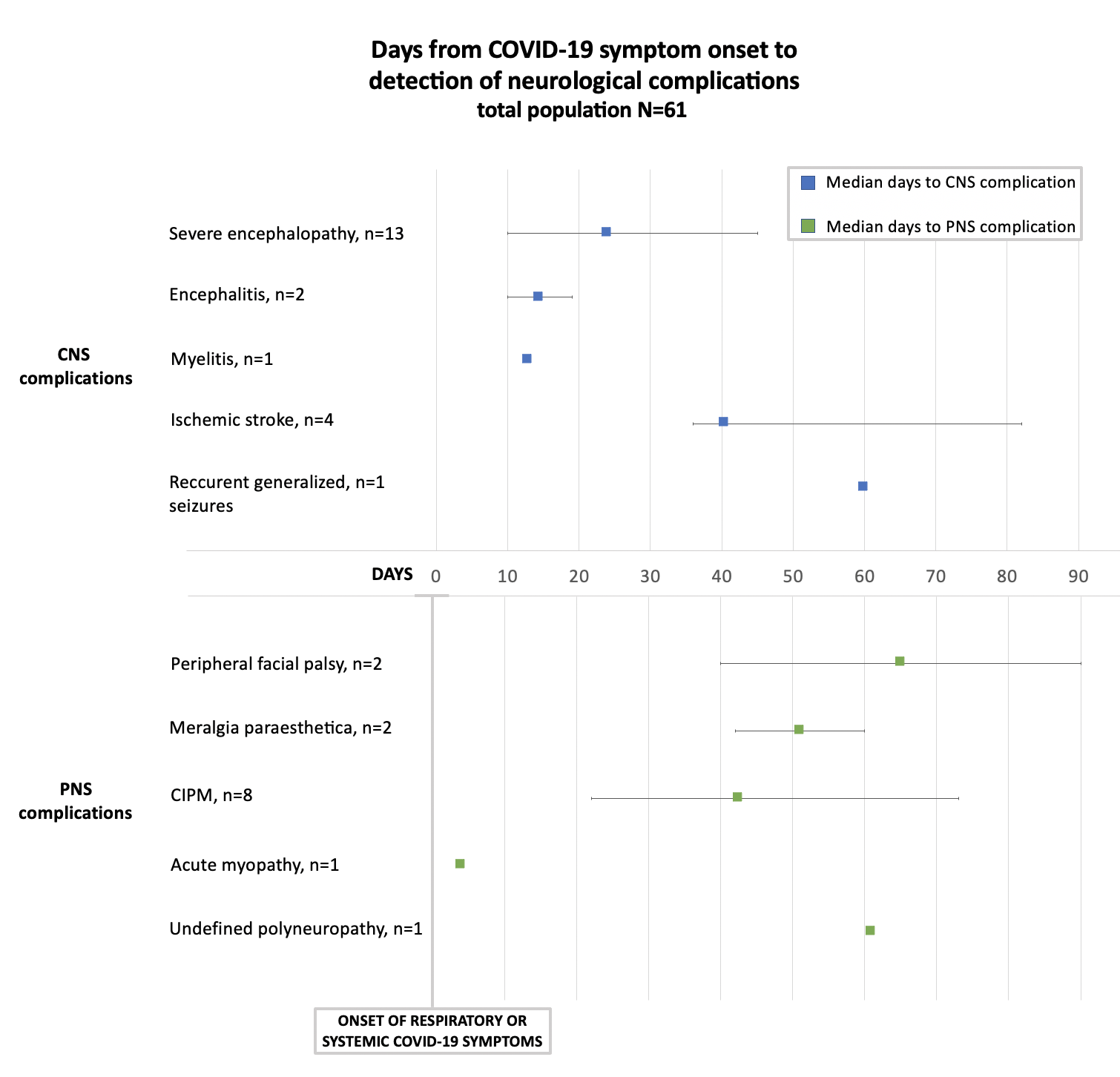

Supplement: Supplementary file 1 — Supplementary file1 (DOCX 839 KB) [file 415_2020_10380_MOESM1_ESM.docx]
